# Mutilation of a Child by a Midwife

**Published:** 1860-01

**Authors:** H. Carpenter


### ARTICLE 6.

### MUTILATION OF A CHILD BY A MIDWIFE.

BY H. CARPENTER, M. D.

I was called on Saturday morning, April the 9th, to see Mrs. L., aged 27, who I found sitting upon a chair in a leaning position, complaining of excrutiating pain in the right Iliac region, and unable to lie down. Tongue coated in the middle and very red at edges and tip. Pulse ninety. Skin dry and hot. Bowels constipated. Urine deficient and very red.—Pregnant with first child and in her eighth month of gestation. The assigned cause of her illness was walking three miles on the previous day. However, upon further examination, I elicited the following facts: After she was taken with slight pain sent for a midwife who came and gave her Tartar emetic, which acted promptly and very violent. Also gave her other medicines, but could not ascertain what. Then she requested an examinetion per vaginum which was agreed to. However before proceeding, she steamed her over a bucket of hot water, after which she introduced her hand into vagina, and during the manipulations she created severe pain, which continued until next morning, consequently became alarmed and sent for me at once. After ascertaining the above particulars, I directed my treatment to arrest premature labor; correct the secretions and allay the increased action of the heart, for which I gave a large dose of Morphia with Hyd. cum creta. This relieved her so much that she was able to lie down. I then prepared three more powders of the same, directing her to take one every two hours alternately with half teaspoonful of the veratrum mixture, which consists of

Spir. Æther Nit. one drachm.

Tr. Opii. Camph.

Tr. Veratrum Viaieb āā. two drachms.

until she became easy, after which take castor oil. On the 10th her husband came to see me, stated that his wife took the oil after taking two powders—it operated twice—rested well during the night. I gave him several morphia powders to be given as occasion required, requesting him to inform me at once if the pains assumed the character of labor pain.

Friday evening, the 15th, I was called in haste, and on arriving was informed that the child's head had been born about half an hour, and pains very inactive; I consequently inferred the child was dead. I introduced my right index finger into the vagina, hooking it under the arm of the child, and made slight traction, which caused its expulsion. I applied cold water to the thoracic regions for the purpose of producing respiratory movement, which had the desired effect, at least caused it to gasp. Then I removed it a convenient distance from its mother in order that I might try artificial respiration; however discovered that the cord had been severed and bowels protruding, which cut off all hope of the child's recovery. After laying it one side, I endeavored to excite a pain that might expel the placenta, but did not succeed. I introduced my hand and grasped the placenta which came away very readily, but found it badly lacerated. After devoting sufficient time to the comfort of the mother, I commenced an examination of the child, which was in the following condition. Head natural, except badly bruised. Abdomen was lacerated from points corresponding with the crest of Ilium; interior portion of the parietes was pressed into the pelvis, and all the viscera below the diaphragm were lying external to abdomen. Left hip joint was dislocated—head of the femur was thrown upwards on the dorsum ilia; funis was severed about six inches from the body, and had the appearance of having been torn some time, as there was no oozing of blood. During all of this time the action of the heart was perceptible.

I learned from the lady after her recovery, that when the midwife made an examination, she found the head presenting, which she said was a condition attended with a great degree of danger to the mother. After manipulating sometime, she said it was all right and the child would be born soon. However, after awaiting sometime, again endeavored to bring on labor, which explained the whole mystery to me. It is very evident that the woman attempted turning and completely murdered the child. But how is it possible for a child to live in utero one week after having the cord severed? It was not possible for the pains to have severed it, as they were very inactive during the entire process of labor.

[In reply to the question "how is it possible for a child to live in utero one week after having the cord severed?" there can be but one answer, and that is, its utter impossibity. In this case the cord was, without doubt, ruptured, as occasionally happens near the termination of labor, at which time it is liable to be put upon the stretch. The case only illustrates the danger of submitting the parturient process to the direction of ignorant persons, for though even when thus obstructed, nature is usually competent to terminate the labor successfully; yet cases every day occur where the life of the child or the mother, or both, is sacrificed to this too prevalent custom. —*Eds.*]

Lunatics in New York City.—In the want of any reliable census of lunatics in the city or state of New York, Dr. Ranney makes the accurate census returns of Massachusetts, in 1854, the basis of an approximative estimate, and concludes that there must be about 2000 lunatics at this time in New York city.

